# Decreased Survival of Glioma Patients with Astrocytoma Grade IV (Glioblastoma Multiforme) Associated with Long-Term Use of Mobile and Cordless Phones

**DOI:** 10.3390/ijerph111010790

**Published:** 2014-10-16

**Authors:** Michael Carlberg, Lennart Hardell

**Affiliations:** Department of Oncology, University Hospital, Örebro SE-701 85, Sweden; E-Mail: lennart.hardell@orebroll.se

**Keywords:** radiofrequency electromagnetic fields, glioma, survival, mobile phone, cordless phone, astrocytoma

## Abstract

On 31 May 2011 the WHO International Agency for Research on Cancer (IARC) categorised radiofrequency electromagnetic fields (RF-EMFs) from mobile phones, and from other devices that emit similar non-ionising electromagnetic fields, as a Group 2B, *i.e.*, a “possible”, human carcinogen. A causal association would be strengthened if it could be shown that the use of wireless phones has an impact on the survival of glioma patients. We analysed survival of 1678 glioma patients in our 1997–2003 and 2007–2009 case-control studies. Use of wireless phones in the >20 years latency group (time since first use) yielded an increased hazard ratio (HR) = 1.7, 95% confidence interval (CI) = 1.2–2.3 for glioma. For astrocytoma grade IV (glioblastoma multiforme; n = 926) mobile phone use yielded HR = 2.0, 95% CI = 1.4–2.9 and cordless phone use HR = 3.4, 95% CI = 1.04–11 in the same latency category. The hazard ratio for astrocytoma grade IV increased statistically significant per year of latency for wireless phones, HR = 1.020, 95% CI = 1.007–1.033, but not per 100 h cumulative use, HR = 1.002, 95% CI = 0.999–1.005. HR was not statistically significant increased for other types of glioma. Due to the relationship with survival the classification of IARC is strengthened and RF-EMF should be regarded as human carcinogen requiring urgent revision of current exposure guidelines.

## 1. Introduction

### 1.1. Background

The use of both mobile and cordless phones has increased rapidly between the mid-1990s and early 2000s and has since then remained stable at a very high level. During use these devices emit radiofrequency electromagnetic fields (RF-EMFs) and also extremely low frequency electromagnetic fields (ELF-EMFs) from the battery [1,2,3]. The brain is the primary target organ for exposure to electromagnetic fields during the use of handheld phones. This has raised concerns about an increased risk for brain tumours. Many users are children and adolescents, which is of special concern regarding potential health effects on this population.

On 31 May 2011 the WHO International Agency for Research on Cancer (IARC) categorised RF-EMFs from mobile phones, and from other devices that emit similar non-ionising electromagnetic fields, as a Group 2B, *i.e.*, a “possible”, human carcinogen [4,5].

The IARC decision on mobile phones was based mainly on two sets of human case-control studies on brain tumour risk; our studies from Sweden [6,7,8] and the IARC Interphone study and also on available preprint studies [9,10,11]. Both provided complementary and supportive results on positive associations between two types of brain tumours; glioma and acoustic neuroma, and exposure to RF-EMFs from wireless phones.

### 1.2. Some Technical Aspects

The Nordic countries were among the first in the world to widely adopt wireless telecommunications technology. Analogue phones (Nordic Mobile Telephone System—NMT) were introduced in the 1980s using both 450 (1981 to 2007) and 900 (1986 to 2000) Megahertz (MHz) frequencies. The digital system (Global System for Mobile Communication—GSM) using two bands, 900 and 1800 MHz, started to operate in 1991 and now dominates the market. The third generation of mobile phones, 3G or Universal Mobile Telecommunication System (UMTS), using 1900/2100 MHz has been introduced worldwide during the last decade, and in Sweden in 2003. The fourth generation (4G; LTE) was introduced in parts of Sweden at the end of 2009.

Desktop cordless phones have been used in Sweden since 1988, first using the analogue 800–900 MHz frequencies, but since the early 1990s using a digital 1900 MHz system (Digital Enhanced Cordless Telecommunications—DECT). These phones also emit RF-EMF radiation similar to that of mobile phones. Thus, it is necessary to consider the usage of cordless phones, along with mobile phones, when human health risks are evaluated.

### 1.3. Aim of the Study

The aim of this study was to analyse survival of glioma patients in our case-control studies in relation to use of wireless phones; mobile and cordless phones [6,8,12,13]. Survival was assessed using the Swedish population registry that is continuously up-dated. All inhabitants can be followed using the unique ID number for each subject. If deceased, date of death is recorded. All cases were followed from date of diagnosis based on the histopathology report until 18 December 2013 or if deceased, date of death. We have previously reported survival for patients with glioma in our case-control study for the time period 1997–2003 [14]. We have now up-dated these results with further follow-up of these cases and furthermore added our study for the time period 2007–2009 [13]. This publication covers both study periods with up-dated results on survival. It should be noted that all diagnoses were based on histopathology. Thus, all cases had undergone some type of surgery.

## 2. Materials and Methods

### 2.1. Study Design

Detailed information on materials and methods in our case-control studies has been given in our publications. In short, six administrative regions with oncology centres covering Sweden register new cancer cases. For 1997–2003, cases and controls covered central Sweden and all were alive at the time of inclusion in the study [6]. We performed in addition a similar case-control study on deceased cases during the study period 1997–2003, using deceased persons as reference entities by interviewing the next of kin of both cases and controls [12]. The 2007–2009 study included the whole country and encompassed only living cases [13]. The oncology centres reported new cases diagnosed during these years with histopathology verified brain tumours, either benign or malignant, to us. Both men and women aged 20–80 years (1997–2003) and 18–75 years (2007–2009) at the time of diagnosis were included. Tumour localisation in the brain was based on reports to the cancer registries and medical records.

Controls were ascertained from the Swedish Population Registry, covering the whole country and continuously updated. Each person could be traced by the personal ID number. This study includes only the cases.

Exposure was assessed using a mailed questionnaire sent to each person. Use of mobile phones and cordless desktop phones was covered by questions on first year of use, total number of years, average daily use, use of a hands-free device, and preferred ear (for further details see [6,12,13]). The procedure was conducted without knowledge of case or control status. Use of mobile and cordless phones was referred to as ipsilateral (≥50% of the time) or contralateral (<50% of the time) in relation to tumour side.

A number of questions regarding other potential risk factors for brain tumours were also included in the questionnaire. If the answers in the questionnaire were unclear, they were resolved by phone using trained interviewers. Each questionnaire had received a unique ID-number that did not disclose whether it was a case or a control; *i.e.*, the interviewer was unaware of the status and the same applied to the further data processing. All information was coded and entered into a database. Case or control status was not disclosed until statistical analyses were undertaken.

The study was conducted in accordance with the Declaration of Helsinki, and the protocol was approved by the Ethics Committee of Örebro University Hospital (Dnr 351/96, 9 April 1996 and 9/02, 6 May 2002), and Uppsala University (Dnr 2005:367, 22 February 2006). Informed consent for inclusion was obtained before participation.

### 2.2. Statistical Methods

StataSE 12.1 (Stata/SE 12.1 for Windows; StataCorp., College Station, TX, USA) was used for all analyses. Wilcoxon rank-sum test was used for calculation of *p*-values for comparisons of age between exposed and unexposed to wireless phones. The Cox proportional hazards model was used to calculate hazard ratios (HR) and corresponding 95% confidence intervals (CI). Follow-up time was counted from the date of diagnosis (defined as the date of the histopathology report) to the date of death or 18 December 2013 (living cases). Adjustment was made for age (as a continuous variable), gender, year of diagnosis, socioeconomic (SEI)-code and study (material with living cases interviewed and material with next-of-kin interviewed). The proportional hazards assumption was tested using Schoenfeld residuals. A statistically significant violation of the proportionality assumption was detected for age; therefore age was also adjusted for as a time-dependent covariate.

The exposed cases were divided according to phone type; analogue, digital, and cordless. The use of analogue and digital phones was analysed combined (*i.e.*, mobile phone) and results for all phone types combined (mobile and/or cordless phone = wireless phone) are also presented. Note that some cases could have used both mobile and cordless phones. The unexposed category consisted of cases that reported no use of wireless phones, or only exposure ≤1 year before diagnosis.

Latency (time from first use) was analysed using five time periods, >1–5 years, >5–10 years, >10–15 years, >15–20 years and >20 years since first use of a mobile or a cordless phone until diagnosis. Cumulative use of the phone types was analysed in quartiles based on the distribution of total use of wireless phones among the controls; first quartile 1–122 h; second quartile 123–496 h; third quartile 497–1460 h, fourth quartile >1460 h. Note that since some cases have used both a mobile and a cordless phones they may be included in a higher quartile category of cumulative wireless phone use than for each phone type separately. Latency and cumulative use were also analysed as continuous variables (per year of latency, per 100 h cumulative use).

Restricted cubic splines were used to display the relationship between cumulative use and latency of wireless phones with astrocytoma grade IV survival. Adjustment was made for the same variables as in the Cox proportional hazards model. Four knots were used at the 5th, 35th, 65 and 95th percentiles, as suggested by Harrell [15]. *p*-values for non-linearity were estimated by testing whether the coefficient of the second and third spline was equal to zero by using the Wald test.

## 3. Results

### 3.1. Overall Results

In total 1678 cases with glioma were included in this study. This constituted 91% of all cases with a malignant brain tumours (n = 1844; 86% response rate). For 16 cases diagnosis was based on autopsy so they were excluded from this analysis (survival 0 days).

Statistics of the age distribution are shown in Table 1 both for glioma cases exposed to wireless phones and for unexposed subjects. The median age for exposed cases was 54 years (range 19–80 years) and for unexposed cases 63 years (range 21–80 years), *p* < 0.0001. Of the glioma cases 322 (19%) were alive at 18 December 2013; 24 of astrocytoma grade IV (glioblastoma multiforme) cases (3%).

### 3.2. All Glioma

Survival decreased for glioma cases per year of latency for mobile phone use, HR = 1.016, 95% CI = 1.004–1.029, Table 2. Also for cordless phone use an increased HR was found, although it was not statistically significant. Wireless phone use in total yielded HR = 1.015, 95% CI = 1.003–1.026 per year of latency. Regarding cumulative use per 100 h, cordless phones yielded increased HR of borderline significance, whereas HR per 100 h cumulative use of mobile phones was not changed from unity.

We calculated HR and 95% CI in different latency groups. In the longest latency group >20 years mobile phone use gave for all glioma HR = 1.8, 95% CI = 1.3–2.5 (*p*, trend = 0.01), cordless phone use HR = 1.3, 95% CI = 0.5–3.7 (*p*, trend = 0.08) and wireless phone use in total HR = 1.7, 95% CI = 1.2–2.3 (*p*, trend = 0.01) for decreased survival, Table 3.

Analysis of glioma cases with latency >20 years and cumulative use >1460 h (fourth quartile) gave for wireless phone use HR = 2.2, 95% CI= 1.4–3.3 (n = 56, data not in Table). Thus, HR was slightly higher than in the group of >20 years in the separate latency analysis (Table 3).

### 3.3. Low-Grade Astrocytoma

The median survival of the 228 cases with low-grade astrocytoma (grade I–II) was 3102 days (8.5 years). Statistically significant decreased HR (increased survival) was found for mobile phone use; HR = 0.5, 95% CI = 0.3–0.9, cordless phone use non-statistically significant HR = 0.6, 95% CI = 0.3–1.1 and for wireless phones in total, statistically significant decreased HR = 0.6, 95% CI = 0.3–0.9, Table 2. However, HR was not statistically significant per 100 hours of cumulative use or per year of latency (Table 2). Lower HR than unity was calculated in the different latency groups and quartiles of cumulative use, but there was no statistically significant trend for any phone type (data not in Table 3).

### 3.4. Astrocytoma Grade III

The median survival of the 196 cases with astrocytoma grade III (anaplastic) was 562 days (1.5 years). HR was somewhat decreased in total for mobile phone use, HR = 0.7, 95% CI = 0.5–1.2 and cordless phone use, HR = 0.7, 95% CI = 0.4–1.2. HR was not statistically significant increased or decreased per year of latency or per 100 h cumulative use, Table 2. For both phone types there was no statistically significant trend for the different latency groups or cumulative use in quartiles (data not in Table 3).

### 3.5. Astrocytoma Grade IV

The median survival of the 926 cases with astrocytoma grade IV (glioblastoma multiforme) was 344 days (0.9 year). The median age of cases with astrocytoma grade IV (glioblastoma multiforme) was higher than for other glioma cases (Table 1). Furthermore, exposed cases had lower median age than unexposed cases, 59 years *versus* 66 years, *p* < 0.0001. A decreased survival was found per year of latency for mobile phone use, HR = 1.017, 95% CI = 1.004–1.031, and for cordless phone use, HR = 1.023, 95% CI = 1.004–1.043, Table 2. HR increased statistically significant per year of latency for wireless phone use in total. HR was slightly increased per 100 h cumulative use of both mobile and cordless phones, although not statistically significant.

In Table 3 the risk of decreased survival in different latency groups is displayed. The longest latency, >20 years, gave HR = 2.0, 95% CI = 1.4–2.9 for mobile phone use (*p*, trend = 0.02) and HR = 3.4, 95% CI = 1.04–11 for cordless phone use (*p*, trend = 0.07). Wireless phone use in total gave HR = 2.1, 95% CI = 1.5–3.0 (*p*, trend = 0.01). OR was increased in the fourth quartile of cumulative use, >1460 h, both for mobile and cordless phones although not statistically significant and without a statistically significant trend.

Analysis of glioma cases with astrocytoma grade IV in the latency >20 years and cumulative use >1460 h (fourth quartile) group gave for wireless phone use HR = 1.8, 95% CI = 1.1–2.9 (n = 37, data not in Table 3). Thus, HR was similar as in the group of >20 years in the separate analysis of latency (Table 3). We analysed also survival for cases in different age groups of first use of the wireless phone, Table 3. HR was highest for cases with first use of both mobile and cordless phones before the age of 20. Wireless phone use in total gave HR = 2.3, 95% CI = 1.1–4.7 in that age group.

### 3.6. Oligodendroglioma

The median survival of the 171 cases with oligodendroglioma was 3288 days (9.0 years). HR was not statistically significant increased per year of latency or per 100 h cumulative use, Table 2. In the longest latency group >20 years, mobile phone use gave HR = 4.3, 95% CI = 1.01–18 (n = 7 cases; *p*, trend = 0.08). Only one case reported cordless phone use in that latency group (*p*, trend = 0.23). Wireless phone use with >20 years latency gave HR = 2.5, 95% CI = 0.6–10 (n = 8 exposed cases, *p*, trend = 0.04; data not in Table 3). No statistically significant trend was found for cumulative use in quartiles of mobile or cordless phone use.

### 3.7. Other/Mixed Glioma

The median survival of the 154 cases with other or mixed glioma was 2285 days (6.3 years). HR increased per year of latency for wireless phone use, although not statistically significant, HR = 1.048, 95% CI = 0.993–1.107. Cumulative use of wireless phones produced HR = 1.000, 95% CI = 0.993–1.008 (Table 2). Increased HR was calculated in the >20 years latency group for mobile and cordless phone use, although with no statistically significant trend. Wireless phone use in the same latency group produced HR = 2.7, 95% CI = 0.6–12 (n = 8 exposed cases, *p*, trend = 0.16; data not in Table 3).

### 3.8. Restricted Cubic Spline Plots

**Figure 1:**
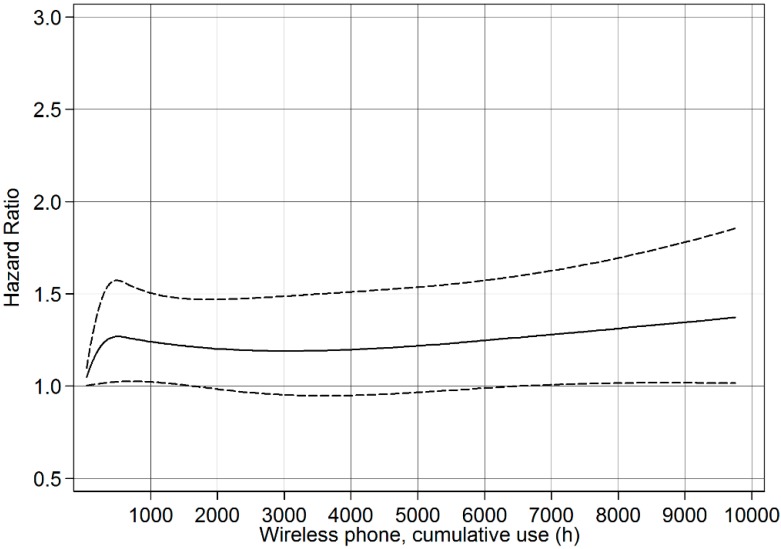
Restricted cubic spline plot of the relationship between cumulative use of wireless phones and astrocytoma grade IV. The solid line indicates the HR estimate and the broken lines represent the 95% CI. Adjustment was made for age, gender, year of diagnosis, SEI-code, study, and age as a time-dependent covariate.

**Figure 2:**
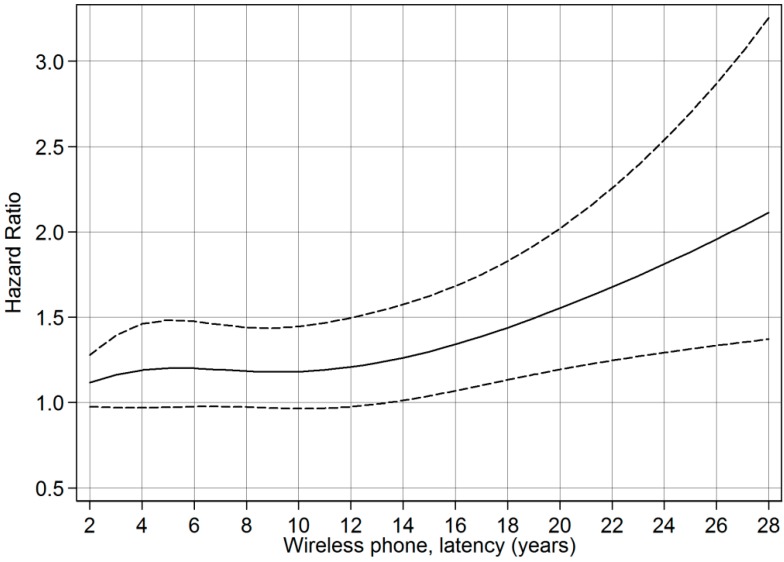
Restricted cubic spline plot of the relationship between latency of wireless phones and astrocytoma grade IV. The solid line indicates the HR estimate and the broken lines represent the 95% CI. Adjustment was made for age, gender, year of diagnosis, SEI-code, study, and age as a time-dependent covariate.

The restricted cubic spline plot of the HR between cumulative use of wireless phones and survival of cases with astrocytoma grade IV is displayed in Figure 1. There was a linear trend of increasing HR up to 10000 h of cumulative use (non-linearity, *p* = 0.12). According to Figure 1 the result was of borderline statistical significance for cumulative use > 6000 h. A linear relationship between latency of wireless phone use and HR was also calculated, Figure 2 (non-linearity, *p* = 0.34). Statistically significant hazard ratios were found for latencies ≥14 years.

**Table 1:** Statistics of the age distribution for all cases and for exposed/unexposed to wireless phones.

	Exposed Wireless Phone					Unexposed Wireless Phone					*p* *	All				
	n	Mean	Median	Min	Max	n	Mean	Median	Min	Max		n	Mean	Median	Min	Max
Glioma	1198	52	54	19	80	480	61	63	21	80	<0.0001	1678	54	57	19	80
-Astrocytoma	951	53	56	19	80	402	62	64	23	80	<0.0001	1353	56	58	19	80
-grade I–II	189	41	39	19	74	39	48	52	23	71	0.02	228	42	40	19	74
-grade III **	143	48	50	20	80	53	60	63	27	77	<0.0001	196	51	54	20	80
-grade IV **	618	58	59	21	79	308	64	66	29	80	<0.0001	926	60	61	21	80
-Oligodendroglioma	135	48	48	20	73	36	54	57	22	75	0.02	171	49	50	20	75
-Other/mixed glioma	112	48	47	20	75	42	50	54	21	79	0.33	154	48	48	20	79

Notes: * Wilcoxon rank-sum test; ** Information regarding grade III or IV missing for 3 cases.

**Table 2:** Hazard ratio (HR) and 95% confidence interval (CI) from the Cox proportional hazards model for glioma and subtypes and use of mobile and cordless phones, overall results (ever use), per 100 h cumulative use, and per year of latency. Adjustment was made for age, gender, year of diagnosis, SEI-code, study, and age as a time-dependent covariate.

	Mobile Phone				Cordless Phone				Wireless Phone			
	n, exp	HR	95% CI	*p*	n, exp	HR	95% CI	*p*	n, exp	HR	95% CI	*p*
**Glioma, all** **(n_tot_ = 1678; n_unexp_ = 480)**												
All	1037	1.1	0.9–1.3	0.37	825	1.0	0.9–1.2	0.85	1198	1.1	0.9–1.2	0.32
-Per 100 h cumulative use		1.000	0.997–1.003	0.97		1.004	1.0002–1.009	0.04		1.001	0.999–1.003	0.37
-Per year of latency		1.016	1.004–1.028	0.01		1.014	0.997–1.030	0.10		1.015	1.003–1.026	0.01
**Astrocytoma, grade I–II** **(n_tot_ = 228; n_unexp_ = 39)**												
All	164	0.5	0.3–0.9	0.02	135	0.6	0.3–1.1	0.09	189	0.6	0.3–0.9	0.03
-Per 100 h cumulative use		0.996	0.982–1.009	0.52		0.990	0.974–1.006	0.21		0.995	0.986–1.005	0.32
-Per year of latency		0.966	0.913–1.022	0.23		0.964	0.906–1.027	0.26		0.966	0.920–1.016	0.18
Astrocytoma, grade III * **(n_tot_ = 196; n_unexp_ = 53)**												
All	127	0.7	0.5–1.2	0.22	95	0.7	0.4–1.2	0.16	143	0.8	0.5–1.3	0.38
-Per 100 h cumulative use		1.000	0.993–1.007	0.95		1.011	0.995–1.028	0.18		1.000	0.995–1.006	0.87
-Per year of latency		0.988	0.953–1.025	0.54		0.991	0.943–1.042	0.73		0.988	0.955–1.023	0.49
Astrocytoma, grade IV * **(n_tot_ = 926; n_unexp_ = 308)**												
All	532	1.2	0.95–1.4	0.15	415	1.1	0.9–1.4	0.20	618	1.2	0.98–1.4	0.08
-Per 100 h cumulative use		1.002	0.998–1.006	0.30		1.004	0.998–1.010	0.18		1.002	0.999–1.005	0.14
-Per year of latency		1.017	1.004–1.031	0.01		1.023	1.004–1.043	0.02		1.020	1.007–1.033	0.003
**Oligodendroglioma** **(n_tot_ = 171; n_unexp_ = 36)**												
All	119	1.0	0.5–2.1	0.92	99	0.6	0.2–1.3	0.17	135	1.1	0.6–2.2	0.72
-Per 100 h cumulative use		0.992	0.969–1.016	0.52		0.988	0.952–1.026	0.55		0.992	0.975–1.010	0.40
-Per year of latency		1.000	0.947–1.057	0.996		0.917	0.837–1.006	0.07		0.973	0.922–1.026	0.31
**Other/mixed glioma** **(n_tot_ = 154; n_unexp_ = 42)**												
All	95	1.7	0.9–3.4	0.12	80	1.7	0.9–3.3	0.10	112	1.7	0.9–3.1	0.11
-Per 100 h cumulative use		0.996	0.984–1.008	0.54		1.010	0.997–1.023	0.13		1.000	0.993–1.008	0.90
-Per year of latency		1.039	0.984–1.098	0.17		1.021	0.943–1.106	0.61		1.048	0.993–1.107	0.09

Note: * Information regarding grade III or IV missing for 3 cases.

**Table 3:** Hazard ratio (HR) and 95% confidence interval (CI) from the Cox proportional hazards model for all glioma and for astrocytoma grade IV and use of mobile and cordless phones in different latency groups, quartiles of cumulative use, and age at first use. Adjustment was made for age, gender, year of diagnosis, SEI-code, study, and age as a time-dependent covariate.

	Mobile Phone				Cordless Phone				Wireless Phone			
	n, exp	HR	95% CI	*p*	n, exp	HR	95% CI	*p*	n, exp	HR	95% CI	*p*
**Glioma, all** **(n_tot_ = 1,678; n_unexp_ = 480)**												
Latency *												
>1–5 year	291	1.0	0.8–1.2	0.97	299	0.9	0.8–1.1	0.35	307	1.0	0.8–1.2	0.90
>5–10 year	331	1.2	0.95–1.4	0.15	328	1.1	0.9–1.4	0.18	399	1.2	0.99–1.4	0.06
>10–15 year	229	1.1	0.9–1.4	0.45	142	1.2	0.9–1.5	0.21	273	1.1	0.9–1.4	0.32
>15–20 year	107	1.2	0.9–1.6	0.19	50	0.9	0.6–1.3	0.53	136	1.1	0.8–1.4	0.54
>20 year	79	1.8	1.3–2.5	<0.001	6	1.3	0.5–3.7	0.56	83	1.7	1.2–2.3	0.001
Hours *												
First quartile **	428	1.0	0.9–1.2	0.66	190	0.9	0.7–1.1	0.19	290	1.0	0.9–1.2	0.92
Second quartile	203	1.1	0.9–1.4	0.25	218	1.0	0.8–1.2	0.80	260	1.1	0.9–1.3	0.44
Third quartile	181	1.3	0.99–1.6	0.06	233	1.2	0.95–1.5	0.13	261	1.2	0.98–1.5	0.08
Fourth quartile	225	1.1	0.9–1.4	0.41	184	1.3	0.99–1.6	0.06	387	1.2	0.97–1.4	0.09
**Age, first use**												
<20 years old	69	1.0	0.6–1.7	0.97	46	0.9	0.5–1.7	0.83	81	1.0	0.6–1.6	0.91
20–49 years old	646	1.1	0.9–1.4	0.16	457	1.0	0.8–1.3	0.72	724	1.1	0.9–1.3	0.23
≥50 years old	322	1.0	0.9–1.2	0.81	322	1.0	0.8–1.2	0.96	393	1.0	0.9–1.2	0.54
**Astrocytoma, grade IV** **(n_tot_ = 926; n_unexp_ = 308)**												
Latency ***												
>1–5 year	137	1.2	0.9–1.5	0.18	137	1.0	0.8–1.3	0.76	145	1.1	0.9–1.4	0.24
>5–10 year	157	1.1	0.9–1.4	0.47	174	1.2	0.9–1.5	0.14	201	1.1	0.9–1.4	0.24
>10–15 year	120	1.2	0.9–1.6	0.17	77	1.5	1.1–2.1	0.01	142	1.3	1.01–1.7	0.04
>15–20 year	66	1.2	0.8–1.6	0.35	24	1.2	0.7–2.0	0.43	76	1.3	0.9–1.7	0.14
>20 year	52	2.0	1.4–2.9	<0.001	3	3.4	1.04–11	0.04	54	2.1	1.5–3.0	<0.001
Hours ***												
First quartile **	228	1.1	0.9–1.4	0.25	97	0.9	0.7–1.2	0.67	159	1.1	0.9–1.3	0.54
Second quartile	105	1.1	0.9–1.5	0.32	97	1.1	0.9–1.5	0.36	129	1.2	0.9–1.5	0.21
Third quartile	87	1.4	1.01–1.8	0.045	121	1.4	1.04–1.8	0.02	140	1.3	1.1–1.7	0.02
Fourth quartile	112	1.2	0.9–1.6	0.34	100	1.2	0.9–1.6	0.12	190	1.3	0.99–1.6	0.06
**Age, first use**												
<20 years old	10	2.2	1.04–4.8	0.04	6	1.8	0.7– 4.7	0.24	11	2.3	1.1–4.7	0.03
20–49 years old	296	1.2	0.98–1.6	0.07	177	1.3	1.001–1.7	0.049	328	1.2	0.99–1.5	0.07
≥50 years old	226	1.1	0.9–1.4	0.31	232	1.1	0.9–1.3	0.43	279	1.1	0.9–1.4	0.17

Notes: * *p*, trend: Mobile phone: latency *p* = 0.01; hours *p* = 0.36; Cordless phone: latency *p* = 0.08; hours *p* = 0.01; Wireless phone: latency *p* = 0.01; hours *p* = 0.29. ** First quartile 1–122 h; second quartile 123–496 h; third quartile 497–1460 h, fourth quartile > 1460 h; *** *p*, trend: Mobile phone: latency *p* = 0.02; hours *p* = 0.58; Cordless phone: latency *p* = 0.07; hours *p* = 0.09; Wireless phone: latency *p* = 0.01; hours *p* = 0.28.

## 4. Discussion

The main finding in this enlarged study compared with our previous one [14] was decreased survival for cases with glioma associated with long-term use of wireless phones based on a larger material including the study period 2007–2009 and with longer follow-up of cases diagnosed 1997–2003. HR increased with latency as in our first publication. Regarding cumulative use there was no statistically significant trend, except for cordless phones with increased HR per 100 h cumulative use. Fourth quartile of wireless phone use, >1460 h, gave somewhat increased although not statistically significant HR. We did not find any clear evidence of higher HR when combining the group with longest latency (>20 years) with the highest quartile of cumulative use (>1460 h) compared to analyzing latency separately. However, the results were based on low numbers and should be interpreted with caution. According to Hutter *et al.* [16] there is rather poor agreement for intensity of use but better agreement for years of use.

It should be pointed out that by controlling for year of diagnosis and type of study (deceased and living cases at time of inclusion) potential differences in survival during this long period of more than a decade of recruitment are accounted for. Also a potential difference in reporting between self and proxy reports is accounted for in the analysis.

For different types of glioma 95% CI included unity except for astrocytoma grade IV. This is the most malignant glioma type. Most of the glioma cases, 55%, had astrocytoma grade IV. This is a somewhat lower percentage than would be expected, 60%–75%, but is explained by the fact that deceased cases with short survival were not included during the study period 2007–2009 [13]. Most of these tumour types would be astrocytoma grade IV.

Regarding astrocytoma grade IV, the most malignant glioma type, HR increased statistically significant per year of latency for mobile and cordless phone use and wireless phone use in total. The results regarding latency for astrocytoma grade IV are similar as in our previous analysis. In that study the longest latency period was >10 years yielding for wireless phone use HR = 1.3, 95% CI = 1.03–1.7 [14]. With longer latency period, >20 years in the present study, HR increased further to 2.1, 95% CI = 1.5–3.0 indicating worse survival for cases with astrocytoma grade IV with increasing latency period for wireless phone use. In the longer latency period both use of mobile and cordless phones yielded higher HR than in our previous study. These results were verified in the restricted cubic spline plot analysis showing HR clearly increasing with latency.

Like in our previous publication the results for cumulative use were less clear. HR increased slightly per 100 h of cumulative use although not statistically significant. Wireless phone use yielded somewhat increased OR in the fourth quartile, >1460 h, although of borderline statistical significance. In accordance with these results the restricted cubic spline plot showed only slightly increasing trend with cumulative use of wireless phones.

We analyzed survival also for glioma in different anatomical localizations, temporal, frontal or other, without statistically significant findings (data not shown in Table 2 or Table 3). Ipsilateral use of the mobile phone gave slightly increased risk for astrocytoma grade IV, HR = 1.2, 95% CI = 0.96–1.6, contralateral HR = 0.9, 95% CI = 0.7–1.3. Regarding cordless phone use the corresponding results were HR = 1.1, 95% CI = 0.8–1.4 and HR = 0.9, 95% CI = 0.6–1.3, respectively (data not shown).

We have published the highest glioma risk for persons with first use of a wireless phone before the age of 20 compared to older age groups [17]. In this analysis we found that the prognosis was worse in the same age group of first use for patients with astrocytoma grade IV. Thus for persons with first use of mobile or cordless phones before the age of 20 the HR for glioma was higher than for later age groups. In fact HR decreased with increasing age for first use of mobile or cordless phone.Thus, it is notable that subjects with first use before the age of 20 have higher risk to develop astrocytoma grade IV, and they have also worse prognosis than in higher age groups. As we have discussed previously our findings indicate a biological effect from exposure to RF-EMF [14]. The mechanism is unclear although such exposure might change the genetic profile of importance for tumour induction, promotion and prognosis.

The results for other types of glioma are in contrast to the findings for astrocytoma grade IV. Some of the calculations were based on low numbers since the prevalence of these glioma types is clearly lower than for astrocytoma grade IV. Our results indicate no clear effect of RF-EMF exposure on the survival of patients with these tumour types except for low-grade glioma (astrocytoma grade I–II). We found almost identical results as previously based on a larger number, 228 cases now *vs.* 131 previously. Thus wireless phone use in total gave now HR = 0.6, 95% CI = 0.3–0.9 compared with HR = 0.5, 95% CI = 0.3–0.9 previously [14]. Similar results were found for both mobile phone and cordless phone use. The results did not change statistically significant with latency or cumulative use.

The reason for survival benefit for cases with astrocytoma grade I–II associated with use of both mobile and cordless phones is unclear. However, surgery is crucial for survival in patients with low-grade astrocytoma. An earlier treatment gives a better prognosis [18]. Tumour promotion from RF-EMF exposure might give earlier symptoms leading to surgery. We had no data on first symptoms until operation of these patients, which would have been of value in order to compare lag time for exposed and unexposed cases. However, for 144 (63%) of the 228 cases with low-grade astrocytoma it was possible to calculate tumour volume based on CT/MRI scans using the ellipsoid formula:

$$\left(\frac{4}{3}\pi (\frac{D_{1}}{2}\times \frac{D_{2}}{2}\times \frac{D_{3}}{2}\right)$$

where *D*_1_, *D*_2_, *D*_3_ = diameters in the three axis.

This gave for cases exposed to wireless phones (n = 121) median volume = 25.1 cm^3^ (mean = 37.7, range = 0.15–179.6) compared with unexposed cases (n = 23) median volume = 18.3 cm^3^ (mean = 33.1, range = 0.79–125.7). Although the difference was not statistically significant (*p*, Wilcoxon rank-sum test = 0.82) these results indicate tumour promotion since the median tumour volume was 37.2% larger in exposed cases. This might cause tumour awareness and earlier surgery.

Since the low-grade glioma had reached a larger size at time of surgery, another possibility might be that wireless phone users just had surgery at a later, not an earlier, point in tumour development. However this does not explain the better prognosis in that group of patients. Prognosis of low-grade glioma depends strongly on location. If completely resected, which is a function of location, recurrence is infrequent and survival is prolonged. It is possible that wireless phone use causes a different direction of growth that at the same time is related to a slightly different volume but, due to the different location, a better survival. However, due to the low number of low-grade glioma cases with information about tumour volume (n = 144) it was not possible to include location in a meaningful analysis.

Also for astrocytoma grade IV the median tumour volume was larger in exposed cases (n = 346) than in unexposed cases (n = 112), 25.6 *vs.* 22.0 cm^3^ (16.4% larger volume; *p*, Wilcoxon rank-sum test = 0.68). This tumour type is extremely malignant with median survival in the range of 6 months in spite of surgery, radio- and chemotherapy [19]. Thus early detection does not significantly change the prognosis. Median survival was somewhat longer (11 months) in our case series. This is probably explained by the fact that cases that died short after diagnosis were not included during our study period 2007–2009.

In contrast for patients with low-grade glioma long-term survival, even decades, has been reported after surgery for astrocytoma grade I and 6–8 years for astrocytoma grade II. Without surgery, low-grade astrocytoma, especially grade II, may progress to astrocytoma grade IV after 4–5 years, which will determine the prognosis. Also astrocytoma grade III may progress to astrocytoma grade IV without surgery with a mean time of about 2 years. Unfortunately we have no knowledge of cases with low-grade glioma, especially those diagnosed during 1997–2003, that were later diagnosed with a higher grade glioma, which would be of interest.

We had access to histopathology diagnosis for glioma diagnosis, which is a strength of the study. The follow-up of all cases using the Swedish population registry was complete. From that registry date of death can be obtained. For all cases the same criteria for date of diagnosis were used using the date in the Swedish Cancer Registry. Another strength is the high response rate in the assessment of exposure using self-administered questionnaires supplemented over the phone. It is unlikely that the reported use of mobile and cordless phones was related to glioma subtype or prognosis. One disadvantage is that we have no data on use of wireless phones after tumour diagnosis, which would have been of value to get further insight into the mechanism of carcinogenesis from RF-EMF emissions. Of course studies of genetic profile of especially astrocytoma grade IV relating to wireless phone use would be desirable. The *p53* protein is a transcription factor that plays a vital role in regulating cell growth, DNA repair and apoptosis, and *p53* mutations are involved in disease progression. In a recent study it was found that use of mobile phones for ≥3 h a day was associated with increased risk for the mutant type of *p53* gene expression in the peripheral zone of astrocytoma grade IV, and that this increase was statistically significant correlated with shorter overall survival time [20]. The study was rather small (n = 63) and no data on latency of mobile phone use was given.

## 5. Conclusions

The study strengthens the proposed causal association between use of mobile and cordless phones and glioma [21]. Elevated HR (decreased survival) for the most malignant glioma type, astrocytoma grade IV, was found for long-term use of mobile and cordless phones. HR increased slightly for increasing cumulative use. Highest HR was found for cases with first use before the age of 20 years. These results indicate a survival disadvantage for use of wireless phones in that patient group. In contrast decreased HR (improved survival) was found for low-grade astrocytoma indicating survival benefit from wireless phone used. This may be explained by the fact that tumour volume was larger in exposed than in unexposed cases which would cause earlier detection and surgery. Surgery is a determinant for prognosis in this patient group. However, it should be noted that we have reported increased risk for both low-grade (grade I–II) and high-grade astrocytoma (grade III–IV) associated with use of mobile and cordless phones [22].
